# Relationship between Different Psoriasis Types and Thyroid Dysfunction: A Retrospective Analysis

**DOI:** 10.1155/2021/1834556

**Published:** 2021-09-17

**Authors:** Juan Du, Chunyue Ma, Runnan Wang, Lanmei Lin, Luhui Gao, Sunyi Chen, Xiaonian Lu

**Affiliations:** ^1^Department of Dermatology, Huashan Hospital, Fudan University, Shanghai 200040, China; ^2^Department of Oral & Maxillofacial-Head & Neck Oncology, 9th People's Hospital, Shanghai Jiao Tong University School of Medicine, National Clinical Center of Oral Diseases, Shanghai Key Laboratory of Stomatology, Shanghai 200011, China; ^3^Shanghai Medical School, Fudan University, Shanghai 200032, China; ^4^College of Foreign Languages and Literature, Fudan University, Shanghai 200433, China

## Abstract

**Objective:**

The aim of this study was to investigate the relationship between different psoriasis types and thyroid dysfunction.

**Methods:**

The data of patients diagnosed with psoriasis between January 2013 and October 2018 who underwent thyroid function tests were collected. Free triiodothyronine (FT3), free thyroxine (FT4), total triiodothyronine (TT3), total thyroxine (TT4), thyroid-stimulating hormone (TSH), thyroglobulin antibody (TGAb), and thyroid peroxidase antibody (TPOAb) were measured. The thyroid function of patients with psoriasis vulgaris, pustular psoriasis, erythrodermic psoriasis, and psoriatic arthritis was evaluated, and the differences in hormone levels and antibodies in the pituitary-thyroid axis with psoriasis type were analyzed.

**Results:**

The data of a total of 468 patients were analyzed in this study. The proportion of normal hormone levels was higher among vulgaris patients (*P* < 0.001), while the erythrodermic patients were more likely to have decreased FT3 or FT4 but normal TSH (*P* < 0.001). FT3 levels were lower in pustular patients (*P* < 0.05), FT4 levels were lower in erythrodermic patients (*P* < 0.05), and TSH levels were higher in patients with psoriatic arthritis (*P* < 0.05). TPOAb levels were higher than normal in all patients, but there was no significant difference in the levels of TPOAb and TGAb among 4 types of the patients.

**Conclusion:**

Psoriasis is related to thyroid dysfunction, especially in patients with atypical psoriasis types. The possibility of complications should be considered in erythrodermic patients.

## 1. Introduction

Psoriasis is an inflammatory skin disease that is caused by immune dysfunction, determined by the inheritance of multiple genes, and stimulated by many environmental factors. Its prevalence is around 3% worldwide and 0.1–0.5% in China, respectively [1]. This disorder typically affects the skin and joints, but the fingers, toes, and mucous membranes may also be involved. The disease has a chronic and recurrent course. Clinically, psoriasis is broadly divided into 4 types: psoriasis vulgaris, psoriatic arthritis, pustular psoriasis, and erythrodermic psoriasis, of which psoriasis vulgaris is the most common, followed by psoriatic arthritis. The incidence of pustular psoriasis and erythrodermic psoriasis is relatively low, but serious systemic symptoms can occur, affecting the physical and mental health of patients, as well as their quality of life [2].

The precise etiology and pathogenesis of psoriasis have not been fully elucidated. Dysfunction of the innate and acquired immune systems, including activation of the NF-*κ*B signaling pathway for antigen presentation, the dominant response of T helper 1 (Th1) cells, and increased IL-17 response, promoting the development and progression of psoriasis. Thus, patients with psoriasis are at increased risk of other immune system-related diseases, such as rheumatoid arthritis, inflammatory bowel disease, multiple sclerosis, and autoimmune thyroiditis [3, 4]. Hashimoto's thyroiditis, or autoimmune thyroiditis, is a common autoimmune disorder [5]. The T cell-mediated inflammatory response in the thyroid is characterized by high serum levels of TPOAb and TGAb. A dominant Th1 cell response is also present in this disorder, suggesting a possible association between psoriasis and Hashimoto's thyroiditis. In psoriasis, especially psoriatic arthritis, the relationship between autoimmune thyroid damage and thyroid dysfunction remains controversial. There may also be differences in the relationship between adult and juvenile patients as well as sex differences. In this study, the characteristics of thyroid dysfunction in patients with the 4 types of psoriasis were analyzed. The characteristics of autoimmune thyroid damage and pituitary-thyroid axis hormones in each type of patient were also assessed.

## 2. Methods

### 2.1. Patients

The medical data of patients diagnosed with psoriasis between January 2013 and October 2018 who underwent thyroid function tests during admission were retrospectively analyzed. Cases were classified as psoriasis vulgaris, pustular psoriasis, erythrodermic psoriasis, and psoriatic arthritis based on clinical characteristics and pathological examination [6]. Patients with a history of living for a long time in an iodine-deficient region, past history of thyroid disease, history of thyroid surgery or neck radiation, history of systemic application of drugs that may affect thyroid hormone levels, or history of other autoimmune diseases were excluded. Age- and sex-matched subjects, without psoriasis, served as controls.

### 2.2. Thyroid Function Tests

Data on the general characteristics and relevant medical history of the patients were collected, as well as the results of thyroid function tests at admission. The Roche electrochemiluminescence analyzer (Elecsys E170, Roche Diagnostics, Tokyo, Japan) and its supporting reagents (Roche Diagnostics) were used for free triiodothyronine (FT3), free thyroxine (FT4), total triiodothyronine (TT3), total thyroxine (TT4), thyroid-stimulating hormone (TSH), thyroglobulin antibody (TGAb), and thyroid peroxidase antibody (TPOAb) measurements. Reference ranges for normal thyroid function are FT3, 3.50-6.50 pmol/L; FT4, 11.50-22.70 pmol/L; TT3, 1.23-3.39 nmol/L; TT4, 54.0-174.0 nmol/L; TSH, 0.550-4.780 mIU/L; TGAb, <60.0 U/mL; and TPOAb, <60.0 U/mL. Clinical hyperthyroidism was defined as elevated FT3 and FT4 with decreased TSH. Subclinical hyperthyroidism was defined as normal FT3 and FT4 with decreased TSH. Clinical hypothyroidism was defined as decreased FT3 and FT4 with elevated TSH. Subclinical hyperthyroidism was defined as normal FT3 and FT4 with elevated TSH (Table 1).

### 2.3. Ultrasound Tests

Ultrasound examination was carried out using B-mode high-resolution system to measure skin thickness (mm). Normal thickness values were defined as the thickness of the healthy skin surrounding the lesions.

### 2.4. Statistical Analysis

All data were analyzed using SPSS 19.0 software. Measurement data in test results conforming to the normal distribution were expressed as $\bar{x}\pm SD$. Count data were expressed as *n* (%). One-way analysis of variance (ANOVA) and the Kruskal-Wallis tests were used to compare multiple groups for data conforming to the normal distribution and not conforming to the normal distribution, respectively. The *χ*^2^ test was used to compare count data between the groups. Differences with *P* < 0.05 were considered statistically significant.

## 3. Results

### 3.1. Thyroid Function in Patients with Psoriasis

A total of 468 patients were included in this study, including patients with psoriasis vulgaris (*n* = 300), pustular psoriasis (*n* = 60), erythrodermic psoriasis (*n* = 54), and psoriatic arthritis (*n* = 54). There were 318 male and 150 female patients, and the average age was 48.08 ± 15.76 years. There were no statistically significant differences in age or sex among the patients with each disease type (*P* > 0.05).

No cases of clinical hyperthyroidism were found in the patients; however, 37 (7.91%) of patients had abnormal thyroid function, suggesting a possible correlation between psoriasis and thyroid dysfunction. A condition of subclinical hyperthyroidism was found in 25 patients (25/468, 5.3%), which included 13 cases of vulgaris, 5 pustular, 4 erythrodermic, and 3 arthritis type. There was only one patient of clinical hypothyroidism in each of the erythrodermic group and arthritis group. A condition of subclinical hypothyroidism was found in 12 patients (12/468, 2.56%), including 9 cases of vulgaris, 1 arthritis, and 2 pustular subsets (Table 2).

After laboratory tests for thyroid function, 74.2% (347/468) of patients had normal levels of FT3, FT4, and TSH. The proportion of patients with normal FT3, FT4, and TSH was 80.3% (241), 66.7% (40), 48.2% (26), and 74.1% (40), respectively, in vulgaris, pustular, erythrodermic, and arthritis subsets. There was a statistically significant difference among the 4 psoriasis types in the proportion of patients with normal FT3, FT4, and TSH (*χ*^2^ = 26.781, *P* < 0.001). Post hoc analysis between the groups showed a higher proportion of patients with normal hormone levels in the vulgaris group and a higher proportion of patients with abnormal hormone levels in the erythrodermic group. There was a statistically significant difference among the 4 groups in the proportion of patients with decreased FT3 or FT4 but normal TSH (*χ*^2^ = 29.816, *P* < 0.001). Post hoc testing between the groups showed that this abnormality was more likely in the erythrodermic group than in the other 3 groups. Moreover, no statistically significant differences were observed among the 4 groups in the proportion of patients with normal FT3 and FT4 but decreased TSH (*χ*^2^ = 2.126, *P* = 0.547).

### 3.2. Levels of Pituitary-Thyroid Axis Hormone, TPOAb, and TGAb in Psoriasis Patients

Table 3 shows the FT3, FT4, TSH, TT3, and TT4 levels in patients with the 4 psoriasis types. FT3 levels were lower in pustular psoriasis patients, with a statistically significant difference compared to those in psoriasis vulgaris and erythrodermic psoriasis patients (*P* = 0.040). FT4 levels were lower in erythrodermic psoriasis patients compared to psoriasis vulgaris (*P* < 0.001), pustular psoriasis (*P* = 0.019), and arthritis patients (*P* < 0.001). TSH levels in arthritis patients were higher than those in erythrodermic patients (*P* = 0.049). The average TPOAb level in all groups was higher than normal values (Table 3). There were no significant differences in TPOAb or TGAb levels among the groups (*P* > 0.05).

### 3.3. Correlation of Thyroid Dysfunction with Autoantibody Tests between Psoriasis and Controls

To confirm the correlation of thyroid dysfunction with autoantibody tests, we selected 200 age- and sex-matched subjects without psoriasis as controls. Percentage of thyroid dysfunction was similar between psoriatic patients and controls (Table 4). There were differences between subclinical hyperthyroidism or subclinical hypothyroidism patients and 200 controls in terms of positive autoantibodies.

### 3.4. Ultrasound Tests of Plaque Skin Thickness in Psoriasis Patients

The patients did not routinely undergo ultrasound tests; therefore, only 17 psoriasis patients (including 10 psoriasis vulgaris, 4 erythrodermic psoriasis, and 3 psoriatic arthritis) had ultrasound tests. We showed the plaque skin thickness by ultrasound in Table 5 and found no correlation of skin thickness with TSH, TT3, and TT4.

## 4. Discussion

Psoriasis patients are at a higher risk of other autoimmune diseases. In recent years, the relationship between psoriasis and Hashimoto's thyroiditis-related thyroid dysfunction has gained attention [7]. Hashimoto's thyroiditis can present as mild, short-term hyperthyroidism in the early stages but eventually progresses into hypothyroidism. There are many common pathophysiological mechanisms between psoriasis and Hashimoto's thyroiditis. Studies have shown that IFN-*γ*, Th1 cytokines, CXCL10, IL-23, and Th17 are involved in the pathogenesis of psoriasis. Hashimoto's thyroiditis is a disease also mediated by Th1 cells, in which IFN-*γ* and its associated chemokines, such as CXCL10, play an important role. IL-23 and Th17 levels are elevated in psoriasis and Hashimoto's thyroiditis, suggesting a relationship between the immunological pathogenesis of these diseases [8].

A recent cross-sectional retrospective study showed that Hashimoto's thyroiditis and psoriasis are highly correlated [9], especially in patients with psoriatic arthritis. Antonelli et al. [10] found an increased prevalence of thyroid autoimmunity in patients with psoriatic arthritis. In psoriatic arthritis patients, the rate of TPOAb positivity was significantly higher and similar to that in rheumatoid arthritis patients, an increase which was more pronounced in female patients [11]. However, the prevalence of subclinical hypothyroidism in the entire psoriatic arthritis group was similar to that in the rheumatoid arthritis and control groups, and only in female patients was the prevalence higher. Psoriatic arthritis patients with subclinical hypothyroidism have a longer course of disease and multiple joint involvement compared to psoriatic arthritis patients with normal thyroid function. In addition, Gul et al. [12] observed elevated FT4 levels in patients with psoriasis. However, another recent study has shown that no correlation exists between psoriasis and thyroid autoimmunity. [13] This study showed that the average level of TPOAb in the 4 groups of patients with different types of psoriasis was higher than the normal value, suggesting the presence of autoimmune damage to the thyroid in patients with psoriasis. Thyroid dysfunction existed to varying degrees among the 468 patients in this study, but thyroid function tended to be closer to normal in the psoriasis vulgaris patients compared to the other 3 groups. We found that FT3 levels were lower in pustular psoriasis patients and FT4 levels were lower in erythrodermic psoriasis patients. Although there was no significant change in FT3 and FT4 levels among the psoriatic arthritis patients, their TSH levels were elevated, which is consistent with the results of Antonelli et al. [10] In a study by Zoabi et al. [14], thyroid function tests were performed on 100 psoriasis patients and 54 healthy controls. There was no significant difference in thyroid function between the 2 groups, but TSH levels and positive autoantibody titers were higher in patients with severe psoriasis than in those with mild psoriasis. The reasons for these inconsistent results may be related to differences in the sample size, ethnicity, dietary habits, and living situations and may also be related to the severity and type of disease [15]. To confirm the correlation of between psoriasis and thyroid dysfunction, we selected age- and sex-matched subjects without psoriasis as controls. We found that there were differences between subclinical hyperthyroidism or subclinical hypothyroidism patients and controls in terms of positive autoantibodies.

The present study also found that FT3 or FT4 was decreased in patients with erythrodermic psoriasis, and the proportion of patients with normal TSH was as high as 42.59% [16]. This may be due to diffuse erythema and repeated shedding of scales in erythrodermic psoriasis patients, resulting in the loss of large amounts of water, protein, and other nutrients, causing water and electrolyte disturbances, metabolic abnormalities such as hypoproteinemia, and severe symptoms of systemic wasting. Therefore, patients may develop euthyroid sick syndrome, which presents as low T3 or low T4 syndrome and can be easily misdiagnosed as hypothyroidism. However, these results are yet to be confirmed by clinical data, and no studies on patients with psoriasis combined with euthyroid sick syndrome have been reported in the literature. It is generally believed that in euthyroid sick syndrome, the body compensates by reducing energy consumption while the patient is in a diseased state, indicating a period of serious illness. At this time, exogenous thyroxine replacement therapy alone may be of no benefit. After the disease improves, changes in thyroid hormones should be observed and corresponding adjustments made. Therefore, patients with this type of psoriasis should be properly evaluated prior to treatment.

In summary, psoriasis and autoimmune thyroiditis-related thyroid dysfunction may be related to some extent and is associated with the severity and type of psoriasis. The limitations of this study were that this was a retrospective study, and patients do not routinely undergo ultrasound tests so that there were no results of thyroid B-scan ultrasound, pathology, or other diagnostic examinations. Only 17 psoriasis patients (including 10 psoriasis vulgaris, 4 erythrodermic psoriasis, and 3 psoriatic arthritis) had ultrasound tests. We showed the plaque skin thickness by ultrasound and found no correlation of skin thickness with TSH, TT3, and TT4. To elucidate the correlation between various thyroid function test indicators and psoriasis, prospective studies must be conducted in the future for further confirmation. The dominant response of Th1 cells and the high expression of related inflammatory factors appear in both psoriasis and autoimmune thyroiditis. This may be the immune mechanism common to both [14], but a larger sample size, prospective study basis, and more in-depth mechanistic investigation are required. An emphasis on follow-up assessment of thyroid function in psoriasis patients will be beneficial for the proper treatment of those patients with thyroid dysfunction.

## Figures and Tables

**Table 1:** Baseline patient characteristics.

Characteristics	Total patients (*n* = 468)	Psoriasis vulgaris (*n* = 300)	Pustular psoriasis (*n* = 60)	Erythrodermic psoriasis (*n* = 54)	Psoriatic arthritis (*n* = 54)
Mean age ± SD (y)	48.0 ± 15.7	47.8 ± 15.5	46.6 ± 18.6	51.8 ± 15.8	47.4 ± 13.1
Sex (M/F)	318/150	210/90	33/27	44/10	31/23
Duration of disease (y)	5.5 ± 2.9	7.2 ± 4.6	3.8 ± 1.9	4.1 ± 2.3	6.9 ± 3.0
PASI	30.4 ± 11.5	29.53 ± 10.22	33.90 ± 8.32	45.63 ± 12.13	16.10 ± 8.39

M: male; F; female; PASI: psoriasis area and severity index.

**Table 2:** Thyroid dysfunction of psoriasis patients.

	Psoriasis vulgaris	Pustular psoriasis	Erythrodermic psoriasis	Psoriatic arthritis	Total
Elevated FT3 or FT4 & decreased TSH (%)	0	0	0	0	0
Normal FT3 and FT4 & decreased TSH (%) (adjusted residuals)	13 (4.33%) (-1.3)	5 (8.33%) (1.1)	4 (7.41%) (0.7)	3 (5.56%) (0.1)	25 (5.34%)
Decreased FT3 or FT4 & elevated TSH (%)	0	0	1 (1.85%)	1 (1.85%)	2 (0.43%)
Normal FT3 and FT4 & elevated TSH (%)	9 (3.00%)	2 (3.33%)	0	1 (1.85%)	12 (2.56%)
Decreased FT3 or FT4 & normal TSH (%) (adjusted residuals)	37 (12.33%) (-3.9)	13 (21.67%) (0.9)	23 (42.59%) (5.2)	9 (16.67%) (-0.2)	82 (17.52%)
Total	300 (64.10%)	60 (12.82%)	54 (11.54%)	54 (11.54%)	468

FT3: free triiodothyronine; FT4: free thyroxine; TSH: thyroid-stimulating hormone; TT3: total triiodothyronine; TT4: total thyroxine.

**Table 3:** The levels of pituitary-thyroid axis hormones and autoantibodies in the serum of psoriasis patients.

	Psoriasis vulgaris	Pustular psoriasis	Erythrodermic psoriasis	Psoriatic arthritis
FT3 (pmol/L)	4.67 ± 0.60	4.23 ± 0.73	4.57 ± 0.76	4.45 ± 0.71
FT4 (pmol/L)	14.11 ± 2.23	13.68 ± 2.73	12.17 ± 2.57	14.18 ± 2.39
TSH (mIU/L)	1.89 ± 1.44	1.55 ± 1.12	1.63 ± 1.69	3.69 ± 1.48
TT3 (nmol/L)	1.77 ± 0.40	1.57 ± 0.40	1.76 ± 0.59	1.73 ± 0.43
TT4 (nmol/L)	104.05 ± 22.02	96.29 ± 25.67	81.07 ± 24.06	108.49 ± 22.33
TPOAb (U/mL)	75.72 ± 38.47	78.36 ± 43.73	87.23 ± 36.14	85.32 ± 18.18
TGAb (U/mL)	39.99 ± 27.86	46.55 ± 24.46	29.90 ± 23.45	26.97 ± 8.89

FT3: free triiodothyronine; FT4: free thyroxine; TSH: thyroid-stimulating hormone; TT3: total triiodothyronine; TT4: total thyroxine; TPOAb: thyroid peroxidase antibody; TGAb: thyroglobulin antibody.

**Table 4:** Cases with thyroid dysfunction among study participants.

	Patients (*N* = 468)	Controls (*N* = 200)	*P* value
Subclinical hyperthyroidism	25 (5.3)	8 (4)	0.043
Ab (+)	12 (2.6)	1 (0.5)	0.024
Ab (-)	13 (2.8)	7 (3.5)	NS
Clinical hypothyroidism	2 (0.4)	2 (1)	NS
Ab (+)	0	0	NS
Ab (-)	2 (0.4)	2 (1)	NS
Subclinical hypothyroidism	12 (2.6)	3 (1.5)	0.037
Ab (+)	7 (1.5)	1 (0.5)	0.03
Ab (-)	5 (1.1)	2 (1)	NS

NS: not significant; Ab (+): positive thyroid autoantiodies; Ab (-): negative thyroid autoantibodies. Numbers in parentheses represent percentages.

**Table 5:** Ultrasound tests of skin thickness in psoriasis patients.

	Psoriasis vulgaris (*n* = 10)	Erythrodermic psoriasis (*n* = 3)	Psoriatic arthritis (*n* = 4)
Plaque skin thickness	2.1 ± 0.5	1.9 ± 0.7	2.3 ± 0.6
TSH (mIU/L)	1.89 ± 1.44	1.75 ± 1.34	2.57 ± 1.02
TT3 (nmol/L)	1.36 ± 0.22	1.5 ± 0.80	1.2 ± 0.39
TT4 (nmol/L)	107.1 ± 10.6	79.07 ± 12.05	90.33 ± 26.80

## Data Availability

The datasets used and/or analyzed during the current study are available from the corresponding author on reasonable request.
